# Variant profiling of colorectal adenomas from three patients of two families with *MSH3*-related adenomatous polyposis

**DOI:** 10.1371/journal.pone.0259185

**Published:** 2021-11-29

**Authors:** Claudia Perne, Sophia Peters, Maria Cartolano, Sukanya Horpaopan, Christina Grimm, Janine Altmüller, Anna K. Sommer, Axel M. Hillmer, Holger Thiele, Margarete Odenthal, Gabriela Möslein, Ronja Adam, Sugirthan Sivalingam, Jutta Kirfel, Michal R. Schweiger, Martin Peifer, Isabel Spier, Stefan Aretz

**Affiliations:** 1 Institute of Human Genetics, Medical Faculty, University of Bonn, Bonn, Germany; 2 Center for Hereditary Tumor Syndromes, University Hospital Bonn, Bonn, Germany; 3 Department of Translational Genomics, Center of Integrated Oncology Cologne-Bonn, Medical Faculty, University of Cologne, Cologne, Germany; 4 Center for Molecular Medicine Cologne (CMMC), University of Cologne, Cologne, Germany; 5 Department of Anatomy, Faculty of Medical Science, Naresuan University, Phitsanulok, Thailand; 6 Institute for Translational Epigenetics, Medical Faculty and University Clinic Cologne, University of Cologne, Cologne, Germany; 7 Cologne Center for Genomics (CCG), Faculty of Medicine, University of Cologne, University Hospital Cologne, Cologne, Germany; 8 Berlin Institute of Health at Charité, Core Facility Genomics, Berlin, Germany; 9 Max Delbrück Center for Molecular Medicine in the Helmholtz Association, Berlin, Germany; 10 Institute of Pathology, Faculty of Medicine and University Hospital Cologne, University of Cologne, Cologne, Germany; 11 Zentrum für Hereditäre Tumore, BETHESDA Khs. Duisburg, Duisburg, Germany; 12 Cancer Center Amsterdam, Amsterdam University Medical Centers, Amsterdam, The Netherlands; 13 Core Unit for Bioinformatics Data Analysis, Medical Faculty, University of Bonn, Bonn, Germany; 14 Institute for Genomic Statistics and Bioinformatics, Medical Faculty, University of Bonn, Bonn, Germany; 15 Institute for Medical Biometry, Informatics and Epidemiology, Medical Faculty, University of Bonn, Bonn, Germany; 16 Institute of Pathology, University of Lübeck, Lübeck, Germany; Singapore General Hospital, SINGAPORE

## Abstract

The spectrum of somatic genetic variation in colorectal adenomas caused by biallelic pathogenic germline variants in the *MSH3* gene, was comprehensively analysed to characterise mutational signatures and identify potential driver genes and pathways of *MSH3*-related tumourigenesis. Three patients from two families with *MSH3*-associated polyposis were included. Whole exome sequencing of nine adenomas and matched normal tissue was performed. The amount of somatic variants in the MSH3-deficient adenomas and the pattern of single nucleotide variants (SNVs) was similar to sporadic adenomas, whereas the fraction of small insertions/deletions (indels) (21–42% of all small variants) was significantly higher. Interestingly, pathogenic somatic *APC* variants were found in all but one adenoma. The vast majority (12/13) of these were di-, tetra-, or penta-base pair (bp) deletions. The fraction of *APC* indels was significantly higher than that reported in patients with familial adenomatous polyposis (FAP) (p < 0.01) or in sporadic adenomas (p < 0.0001). In MSH3-deficient adenomas, the occurrence of *APC* indels in a repetitive sequence context was significantly higher than in FAP patients (p < 0.01). In addition, the MSH3-deficient adenomas harboured one to five (recurrent) somatic variants in 13 established or candidate driver genes for early colorectal carcinogenesis, including *ACVR2A* and *ARID* genes. Our data suggest that *MSH3*-related colorectal carcinogenesis seems to follow the classical *APC*-driven pathway. In line with the specific function of MSH3 in the mismatch repair (MMR) system, we identified a characteristic *APC* mutational pattern in MSH3-deficient adenomas, and confirmed further driver genes for colorectal tumourigenesis.

## Introduction

Approximately 3–5% of all colorectal cancer (CRC) cases arise secondary to monogenic inherited tumour predisposition syndromes. These comprise Lynch syndrome (hereditary colon cancer without polyposis; HNPCC; OMIM # 120435), which is caused by heterozygous germline variants in the *EPCAM* gene or in one of four DNA mismatch repair (MMR) genes (*MLH1*, *MSH2*, *MSH6*, *PMS2*) and represents the most common type, and several gastrointestinal polyposis syndromes [1, 2]. The most frequent form of polyposis is adenomatous polyposis, which is characterised by the occurrence of dozens to thousands of adenomas, predominantly in the large intestine, but also in the upper gastrointestinal tract.

Two major inherited monogenic forms of colorectal adenomatous polyposis can be delineated. The first is autosomal dominant familial adenomatous polyposis (FAP, OMIM #175100), which is caused by heterozygous germline variants in the tumour-suppressor gene and Wnt signalling pathway regulator *APC* [3, 4].The second is autosomal recessive *MUTYH*-associated polyposis (MAP, OMIM #608456), which is caused by biallelic germline variants in the base-excision-repair gene *MUTYH* [5].

The introduction of whole exome sequencing (WES) approaches has enabled the identification of further disease subtypes. In particular, these include autosomal dominant Polymerase Proofreading associated polyposis (PPAP), which is caused by specific germline missense variants in the proofreading domain of the polymerase genes *POLE* or *POLD1* [6, 7] and autosomal recessive *NTHL1*-associated polyposis (NAP), which is caused by biallelic germline variants in the base-excision-repair gene *NTHL1* [8, 9].

Research has demonstrated that biallelic germline variants in the MMR genes *MSH2*, *MSH6*, and *PMS2*—a condition termed Constitutional MMR Deficiency (CMMRD) or biallelic MMR deficiency—can also result in an early-onset colorectal adenomatous polyposis phenotype [10]. Recently, we identified biallelic pathogenic germline variants in the MMR gene *MSH3* as the genetic cause of a novel, recessively inherited subtype of colorectal adenomatous polyposis [11]. To date, only two families with *MSH3*-related polyposis have been described.

Interestingly, Lynch syndrome and all genetically clarified adenomatous polyposis syndromes with the exception of FAP are caused by genes involved in DNA repair, which typically lead to mutator phenotypes. The few novel adenomatous subtypes are recent findings, and are rare or even very rare, and thus characterisation of their full tumour spectrum remains incomplete.

In addition, data concerning the molecular steps and specific oncogenic pathways that lead to cancer development and progression remain limited in these syndromes. In general, colorectal tumours arising secondary to pathogenic *MUTYH*, *POLE*, *POLD1*, and *NTHL1* germline variants are microsatellite-stable (MSS), and involve somatic variants in driver genes of classical colon tumourigenesis pathways, such as *APC*, *KRAS*, *PIK3CA*, *FBXW7*, or *TP53*. The somatic mutation spectra encompass single base pair (bp) substitutions and specific mutation types, such as C:G>A:T transversions in MAP, and C:G>T:A transitions in NAP-associated tumours. These patterns reflect the underlying function of the impaired gene, and result—in case of NAP-associated tumours—in the specific mutational signature 30 [9]. In contrast, MSH3-deficient tumours are characterised by high microsatellite instability (MSI) of di- and tetranucleotide repeats (the latter phenomenon is termed Elevated Microsatellite Alterations at Selected Tetranucleotide repeats; EMAST), whereas the MMR deficiency observed in Lynch syndrome-associated tumours tends to comprise high MSI at loci containing mono- and dinucleotide repeats.

To our knowledge, no human or mouse study to date has investigated the whole genetic spectrum of *MSH3* deficient tumours or cell lines. Here, human colorectal adenomas were used to analyse somatic variants in MSH3-deficient tumours in more detail. The specific aim of the present molecular profiling study was to identify potential driver genes of tumourigenesis in the only currently known form of human neoplasia to be caused by biallelic pathogenic *MSH3* germline variants.

## Materials and methods

### Patients and collection of polyps

The present study included three patients with biallelic pathogenic germline variants in *MSH3* (1275.1; 1275.6; 1661.2) from two independent families. Clinical information of these patients is provided in the pedigree in S1 Fig. The index patients of these families participated in a previous exome sequencing study with the aim to uncover further genes with high-penetrance causative germline variants in patients with adenomatous polyposis. Details of the original cohort of 102 unrelated patients (including demographic details and clinical characteristics) are shown in Adam et al. 2016, especially in S2 Table [11]. The inclusion criteria were the presence of at least 20 synchronous, or 40 metachronous, histologically confirmed colorectal adenomas, irrespective of inheritance pattern or extraintestinal lesions. Patients from all parts of Germany were recruited by the Institute of Human Genetics, Bonn, all of them (except one) were of central European origin, confirmed by a principal-component analysis. Affected relatives were informed about the study by the index patient and afterwards asked to participate in the present study. The detailed recruitment process was previously described [12]. The recruitment ranges from August 2006 to November 2010. The study was approved by the local ethics review board (Medical Faculty of the University of Bonn ethics review board no. 224/07), all patients provided written informed consent prior to inclusion. The present research study took place in Bonn and Cologne, Germany.

In two of the three patients investigated in the present study, polyps were obtained from colonoscopies and a hemicolectomy (1275.1) or from a rectosigmoidectomy (1275.6), and were preserved as formalin fixed paraffin embedded (FFPE) samples. In patient 1661.2, polyps were obtained as fresh frozen samples during routine surveillance gastroscopy and colonoscopy. All tissue samples were examined by experienced pathologists, and all polyps were characterised as adenomas. In addition to tissue samples from normal colorectal mucosa, leucocyte-derived DNA was available from patients 1275.1 and 1661.2. Details of polyp location, surgical collection and histology are shown in Table 1.

**Table 1:** Characteristics of colorectal polyps from patients with *MSH3*-related adenomatous polyposis investigated via whole exome sequencing.

patient ID	MSH3 germline mutation	normal tissue	polyp ID	location	histology	grade of dysplasia	tissue preparations	coverage depth	targets being covered 30x (%)	no. of variants	no. of variants—non-silent	TMB*	TMB—non-silent
1275.1	c.1148delA; c.3001-2a>c	leucocyte + normal colorectal mucosa	1275.1–3	left hemicolon	tubular adenoma	low grade	paraffin embedded	134	95.1	214	148	6.0	4.1
			1275.1–4	colon sigmoideum	tubular adenoma	low grade	paraffin embedded	136	93.7	121	97	3.4	2.7
			1275.1-A	colon ascendens	tubulovillous adenoma	low grade	paraffin embedded	66	86.8	50	45	1.4	1.3
1275.6	c.1148delA; c.3001-2a>c	normal colorectal mucosa	1275.6–6	colon sigmoideum	tubulovillous adenoma	high grade	paraffin embedded	134	93.9	60	49	1.7	1.4
			1275.6–8	colon sigmoideum / rectum	tubulovillous adenoma	unknown	paraffin embedded	118	86.7	152	117	4.3	3.3
			1275.6–9	colon sigmoideum / rectum	tubulovillous adenoma	low grade	paraffin embedded	77	71.8	158	126	4.4	3.5
1661.2	c.2319-1g>a; c.2760delC	leucocyte + normal colorectal mucosa	1661.2–2,1	small bowel	serrated adenoma	unknown	fresh frozen	129	94.8	79	63	2.2	1.8
			1661.2–3	rectum	adenoma	unknown	fresh frozen	119	93.8	48	39	1.3	1.1
			1661.2–5,2	unknown	adenoma	unknown	fresh frozen	141	94.5	70	51	2.0	1.4

*TMB = Tumour Mutation Burden.

### DNA extraction

Genomic leucocyte DNA was extracted from peripheral EDTA-anticoagulated blood samples using the standard salting-out procedure. Tumour DNA and DNA from normal colorectal mucosa were extracted from the FFPE and fresh-frozen tissues. Macrodissection was used to select tissue areas with a high tumour cell content for the purpose of nucleic acid extraction. For FFPE material, isolation of genomic DNA was performed post-deparaffinisation using the Maxwell RSC DNA Kit and the Maxwell RSC instrument (Promega, Fitchburg WI, U.S.A.). To reduce FFPE-induced sequencing artifacts, an FFPE repair kit was used (NEBNext FFPE DNA Repair Mix, New England Biolabs) and the DNA fragmentation was performed in a buffered solution. For fresh frozen material, genomic DNA was isolated using the QIAamp DNA Mini Kit (Qiagen, Hilden, Germany). DNA samples were quantified using Nanodrop (ThermoFisher, Waltham MA, U.S.A.).

### Whole-exome sequencing and data processing

Library preparation and whole exome target enrichment was performed using the Agilent SureSelect protocol (Human All Exon, V6). Multiplexed paired-end sequencing was performed on an Illumina HiSeq 2000 platform, in accordance with the manufacturer’s protocol. Germline variation analysis and filtering were performed using the ‚Varbank 2’ GUI and pipeline versions 3.0/3.1 (CCG, University of Cologne, Germany). Reads were mapped to human genome reference build GRCh38 using the BWA-MEM alignment algorithm. Variant calls from GATK HaplotypeCaller [13], Samtools mpileup [14], and Platypus [15] were filtered for high-quality variants (QD>5; ARF>0.25; MQ>50; FS<40; MQRankSum>-5; ReadPosRankSum>-5; passed VQSR filter) rare variants with minor allele frequency (MAF ≤ 0.005, as based on the maximum observed population allele frequency in gnomAD v2) and variants predicted to modify a protein sequence or to impair splicing, as indicated by reduced maximum entropy scores (MaxEntScan).

Somatic substitutions, insertions, and deletions were determined by an in-house cancer genome analysis pipeline [16–18], the details of which are presented in the Supplementary Note of Ref [16]. In brief, the sequencing reads were aligned to the human reference genome NCBI build 37 (NCBI37/hg19) using BWA-MEM (version 0.6.1-r104). Possible PCR-duplicates are then masked and excluded from subsequent analyses. Somatic mutation calling was restricted to those regions with sufficient read coverage (≥ 15x read coverage). The calling of somatic variants was performed by applying a statistical model that takes into account the local sequencing depth, allelic fraction in the tumour, the absence of the variant in the sequencing data of the matched normal tissue, forward-backward biases, and background sequencing errors. The somatic variants are further validated by testing their absence in an in-house database of called variants in 300 normal, non-tumour tissues from healthy controls.

### Copy Number Variant (CNV) detection and verification

Genome-wide single nucleotide polymorphism SNP genotyping was performed using the Infinium Omni2.5–8 v1.5 BeadChip array (Illumina). To identify putative CNVs, the genotyped SNP dataset of each sample was analysed with the QuantiSNP algorithm v.2.2. Here, log2 R ratio (LRR) values and B-allele frequency (BAF) values were used to generate CNV calls. As a measure of confidence, a log Bayes factor (logBF) was computed for each CNV. Called deletions <10 kb, comprised of < 5 probes, with max logBF < 20, and duplications < 20 kb, involved < 7 probes, and/or a max logBF < 30 were removed. To identify somatic copy number alterations (CNA), polyp CNAs were compared to CNVs generated from leucocyte and normal tissue DNA. Somatic CNAs were inspected using GenomeStudio CNV Analysis (Illumina), and checked against the Database of Genomic Variants (DGV), to exclude false positive and common CNAs, respectively. To explore their potential relevance, genes affected by somatic deletions were then investigated via data mining of selected databases.

### Analysis of *APC* variants and repeat sequences

The frequency and distribution of *APC* variants in non-MSH3 driven tumourigenesis were analysed using: 1) the germline *APC* variants in FAP patients listed in the reference locus specific database (LSDB, *www*.*lovd*.*nl/APC*) of the *International Society for Gastrointestinal Hereditary Tumours* (InSiGHT) (4177 variants); and 2) somatic *APC* variants in patients with multiple colorectal adenomas (29 variants) identified in previous in-house studies [19] and 3) somatic *APC* variants in sporadic adenomas (high grade colorectal adenomas = HGCA) [20]. To investigate the sequence context, somatic insertions and deletions (indels) of MSH3-deficient adenomas were inspected visually using the *Integrative Genome Viewer* (IGV). For single bp indels, a repeat sequence context was defined as three or more tandem mononucleotides, and for ≥ 2 bp indels as two or more tandem di- or polynucleotide repeat units, respectively. Using the same criteria, the indels of HGCA [20], and germline and somatic *APC* indels, were inspected for a repetitive sequence context using the software Alamut Visual Version 2.11 (Interactive Biosoftware, Rouen, France).

### Extraction of mutational signatures

The contribution of the catalogued Single Base pair Substitution (SBS) Signatures [21] was estimated using the current version V3 of R package DeconstructSigs (signatures.exome.cosmic.v3.may2019) [22]. The COSMIC signatures used were extracted from exome data only, therefore the “default” normalization was used for the trinucleotide counts as also recommended by the developer of DeconstructSigs. CaMuS [23] was used to estimate the contribution of the small Insertion and Deletion (ID) Signatures [24]. To this end, the reference ID signatures were linearly fitted to the ID mutation spectrum of the polyps. Model selection was applied as described [23] using backward parameter selection. To help with ID signature selection, a curve was then generated displaying the cost associated to removing the catalogued signatures from the model. The polyps ID mutation spectrum was extracted as described above and the matrix of ID counts was generated using SigProfilerMatrixGeneratorR [25].

### Calculation of somatic variant frequencies in non hypermutated tumour samples

The frequency of somatic variants in candidate genes in unselected colorectal tumours was calculated using exome data from the Cancer Genome Atlas (TCGA: https://portal.gdc.cancer.gov/exploration). Somatic variants identified in exome data from colonic (n = 273) and rectal (n = 166) adenocarcinomas were downloaded from the TCGA data portal. To correct the data for the presence of passenger variants, hypermutated tumours (>200 variants; 24% of the tumours) were excluded from the dataset [26].

### Pathway analysis

In silico pathway analysis was performed using the Reactome Knowledgebase (*https*:*//reactome*.*org*) [27]. To evaluate the causative relevance of presumed missense variants, CADD scores were obtained for all missense variants, as described elsewhere [28]. The analysis included all somatic truncating variants; missense variants with a CADD threshold score of ≥ 20 (all of which had a MAF of ≤ 0.1); and inframe indels with an MAF of ≤ 0.1.

### Statistical analysis

Fisher´s exact test was used to determine differences in fractions of indels and single nucleotide variants (SNV´s) of all somatic variants between MSH3-deficient adenomas and HGCA, as well as fractions of *APC*-indels and *APC*-SNV´s between MSH3-deficient adenomas, HGCA, germline and somatic *APC*-variants, and fractions of *APC*-indels lying in repeat sequences between MSH3-deficient adenomas, germline and somatic *APC*-variants. For calculations QuickCalcs by GraphPad Software (www.graphpad.com/quickcalcs/) was used. For all analyses two-tailed t-tests were performed.

## Results

### Characterisation of patients and polyps

Whole exome sequencing was performed on DNA derived from the colorectal polyps of three patients with *MSH3*-related adenomatous polyposis, who were members of two independent families (S1 Fig). The underlying compound heterozygous pathogenic *MSH3* germline variants are shown in Table 1, a graphical display of the gene structure and position of the variants can be found elsewhere [11]. Mean coverage (sequencing depth) of the adenomas was 117x (range: 66–141), and the mean percentage of targets with 30x coverage was 90.1% (range 71.8%-95.1%) (Table 1). All somatic variants were inspected visually, few required removal. In most of these cases, removal was due to read mapping inaccuracies.

The total number of somatic variants (including synonymous variants) for all nine polyps was 952 (735 without silent variants). The mean tumour mutational burden (TMB) was 106 variants per polyp (range 48–214) or 3.0 variants/Mb (range 1.3–6.0) for all variants, and 82 variants per polyp (range 39–148) or 2.3 variants/Mb (range 1.1–4.1) excluding silent variants (Table 1). Polyps 1275.6–8 and 1275.6–9 shared >50% percent of their somatic variants, which is suggestive of a common clonal origin. Although the exact physical proximity of these two polyps could not be determined, they originated from the same hemicolectomy specimen. Therefore, these shared variants were considered only once in the process of filtering for driver genes.

To explore if the DNA derived from normal colorectal mucosa might be prone to somatically acquired mutations, we used the leucocyte DNA as a reference in the two patients where both leucocyte and normal colorectal mucosa was available and identified those mutations present in the normal colorectal mucosa but not in leucocyte DNA. This resulted in 7 acquired variants (patient 1275.1) and 16 acquired variants (patient 1661.2). The TMB in normal colorectal tissue of patients 1275.1 and 1661.2 was 0.2 and 0.42, respectively, and thus, as expected, much lower than in the adenoma tissues.

### Mutational features of MSH3-deficient adenomas

The most frequently observed Single Base Substitution (SBS) in the MSH3-deficient adenomas was the C:G>T:A transition. In all but one adenoma, this represented the vast majority of variants. All other observed substitutions made only a small contribution to the mutational burden (Fig A in S2 Fig). The distribution of the different SBS was consistent with the observations in sporadic adenomas (HGCA) [20] (Fig B in S2 Fig) except for adenoma 1661.2-2-1, where the C:G>A:T transversion was observed considerably more often.

**Fig 1:**
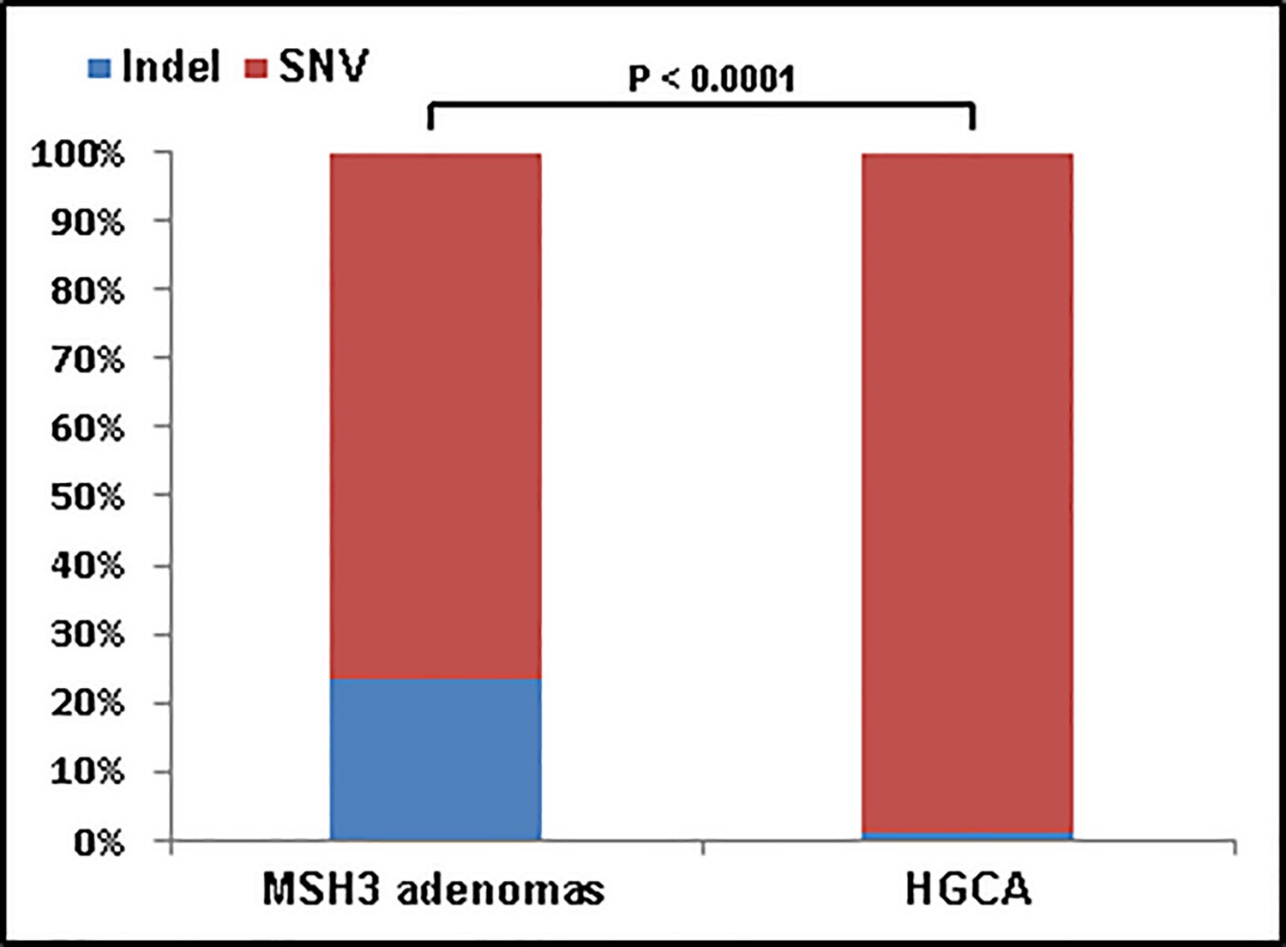
Fractions of indels and SNVs in MSH3-deficient adenomas and HGCA. For all somatic variants of the nine MSH3-deficient adenomas including silent variants (952 variants) and for twelve HGCA (2390 variants), the proportions of indels and SNVs were calculated. In MSH3-deficient adenomas, 24% of all variants were indels, which was significantly higher (p < 0.0001) than in HGCA where the proportion of indels was 1.3%. HGCA = high-grade colon adenoma. Indel = insertion/deletion. SNV = Single Nucleotide Variant.

In the analysis of mutational SBS signatures, signature 1 was found in all nine polyps. In three polyps, SBS 6 contributed with minor effects (S3 Fig 1–9). Although some additional signatures were extracted, these were mainly present in only one or two polyps and contribute with small portions.

Variants were categorised according to their predicted consequences. Analysis of MSH3-deficient adenomas individually revealed that missense variants were the predominant functional type, followed by silent variants. However, in most of the adenomas, a substantial proportion of non-silent variants (mean 31%, range 21%-42%) were indels (S4 Fig). The proportion of indels, i.e. frameshift (FS) and in-frame (IF) variants, and SNVs (including silent variants) for all polyps was calculated, and compared to the respective distributions in HGCA [20]. The proportion of indels in the MSH3-deficient adenomas was significantly (p < 0.0001) higher than in sporadic adenomas (HGCA) (Fig 1).

Subsequently, the indels were separated according to the number of deleted or inserted nucleotides. In the MSH3-deficient adenomas, most indels were 3 or 2 bp in size, although a substantial number of indels affecting 4 bp and a few affecting > 4 bp were also detected (S5 Fig). In HGCA, the vast majority (59%) of indels were 1 bp deletions (S5 Fig) [20]. In the MSH3-deficient adenomas, the majority of the 1–3 bp indels occurred in a repetitive sequence context (S5 Fig). In HGCA, indels occurred more often in a repetitive sequence context than in MSH3-deficient adenomas, although this finding did not reach statistical significance.

**Fig 2:**
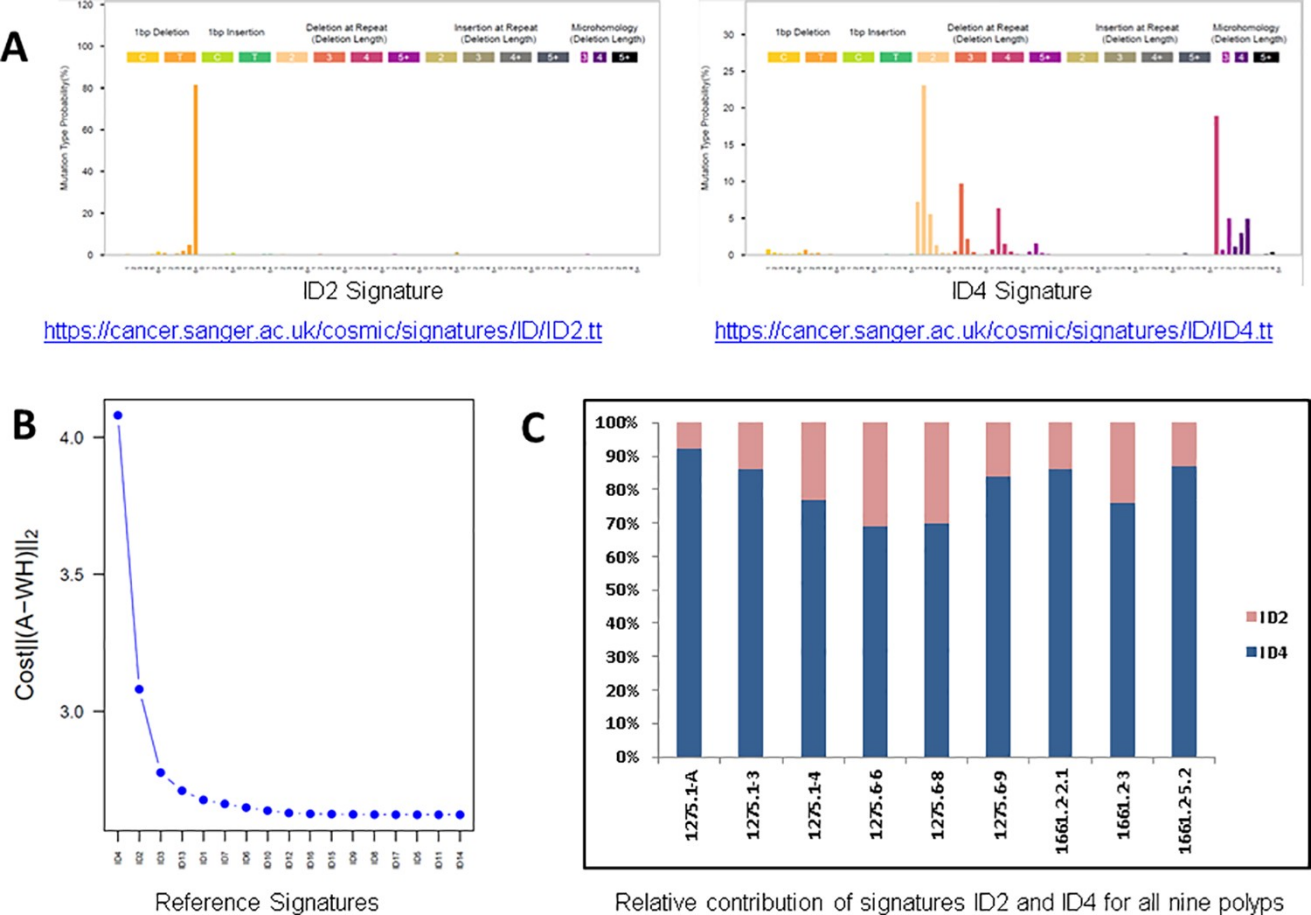
Insertion and deletion mutational signatures of MSH3-deficient adenomas. A) ID2 and ID4 signatures; B) Cost curve used for parameter selection indicating ID2 and ID4 as the signatures mostly influencing the model; and C) The relative contribution of ID signatures 2 (ID2) and 4 (ID4) are displayed for all nine investigated polyps.

The investigation of Small ID signatures revealed that signatures ID2 and ID4 were active in all nine polyps with ID2 showing the greatest contribution (Fig 2). ID2 is characterised by single base pair deletions of thymidine and adenine at a homopolymer length of ≥ 6 bases, and is supposed to represent slippage during DNA replication of the template DNA strand (COSMIC). Signature ID4 is characterised by deletions of mainly 2 bp, but also 3, 4, and ≥ 5 bp deletions, which often occur within units of two repeats. The 2 bp deletions in particular seem to be triggered by small microhomologies at the ID boundaries (COSMIC).

### *APC* variant patterns

Eight of the nine adenomas had truncating somatic variants in *APC*, and five adenomas had two *APC* variants (Table 2, S1 Table). Hence, a total of 13 truncating *APC* variants were identified in the adenomas. The two variants in polyp 1661.2–3 lay in proximity to each other, and analysis of the reads indicated that variants c.4126_4127delTA, and c.4189_4190delGA were in trans, suggesting a loss of function effect for both alleles, consistent with the expected pathomechanism of the tumour suppressor gene (TSG). Of the truncating variants, 46% (6/13) lay within the mutational cluster region (MCR) of the gene. This is very similar to the proportion of around 44% reported for *APC* variants in HGCA [20].

**Table 2:** Most promising (candidate) driver genes of MSH3-related tumourigenesis.

gene	polyp ID	two hits	wild type	mutant	cDNA	protein	mutation type
ACVR2A	1275.1–3		A	-	c.1303delA	p.K435fs	frameshift
	1275.1–4		A	-	c.1303delA	p.K435fs	frameshift
	1275.6–6	x	C	T	c.763C>T	p.R255*	nonsense
	1275.6–6	x	A	-	c.1303delA	p.K435fs	frameshift
APC	1275.1-A		AG	--	c.4385_4386delAG	p.K1462fs	frameshift
	1275.1–4	x	AG	--	c.546_547delAG	p.T182fs	frameshift
	1275.1–4	x	AT	--	c.3629_3630delAT	p.H1210fs	frameshift
	1275.6–6		ACTT	----	c.2800_2803delACTT	p.T934fs	frameshift
	1275.6–8*	x	AG	--	c.730_731delAG	p.R244fs	frameshift
	1275.6–8	x	AG	--	c.4385_4386delAG	p.K1462fs	frameshift
	1661.2–2.1	x	AGTC	----	c.2028_2031delAGTC	p.I676fs	frameshift
	1661.2–2.1	x	AAAAG	-----	c.3921_3925delAAAAG	p.I1307fs	frameshift
	1661.2–3	x, probably trans	TA	--	c.4126_4127delTA	p.Y1376fs	frameshift
	1661.2–3	x, probably trans	GA	--	c.4189_4190delGA	p.E1397fs	frameshift
	1661.2–5.2	x	G	A	c.1659G>A	p.W553*	nonsense
	1661.2–5.2	x	AA	--	c.4382_4383delAA	p.E1461fs	frameshift
ARID1A	1661.2–5.2		AG	--	c.6527_6528delAG	p.Q2176fs	frameshift
ARID1B	1275.6–9		ATT	---	c.6462_6464delATT	p.T2154_L2155delinsT	inframe
ARID2	1275.6–8*		A	-	c.4774delA	p.N1592fs	frameshift
	1661.2–2.1		AG	--	c.2393_2394delAG	p.Q798fs	frameshift
ELF3	1275.6–8*		AGA	---	c.242_244delAGA	p.E81_K82delinsE	inframe
	1661.2–5.2		C	-	c.417delC	p.I139fs	frameshift
FAT4	1275.6–6		A	T	c.1A>T	p.M1L	missense
FBXW7	1275.6–6		G	A	c.1738C>T	p.H580Y	missense
KRAS	1275.6–8		C	A	c.35G>T	p.G12V	missense
LRP5	1661.2–3		G	A	c.1300G>A	p.D434N	missense
MED12	1275.6–6		AGA	--	c.92_94delAGA	p.Q31_K32delinsQ	inframe
SFRP2	1275.6–8*		AGG	--	c.101_103delCCT	p.S34del	frameshift
SYNE1	1275.1–4	x	G	A	c.21436C>T	p.L7146F	missense
	1275.1–4	x	C	A	c.16822G>T	p.E5608*	nonsense
	1275.6–9		C	T	c.25057G>A	p.E8353K	missense
	1661.2–5.2		TC	--	c.8673_8674delGA	p.E2891fs	frameshift
WNT3A	1661.2–3		A	T	c.950A>T	p.N317I	missense

* same variants in 1276.6–8 and 1275.6–9.

Eleven of the twelve *APC* deletion variants were di-, tetra-, or penta-nucleotide deletions (Table 2, S1 Table). The proportion of *APC* indels in the MSH3-deficient adenomas was compared with the fraction of *APC* indels observed among *APC* variants (somatic and germline) from other sources. As we had performed sequencing of *APC* in the adenomas of another patient with *MSH3*-related adenomatous polyposis (sister of 1661.2) in a previous study [19], the eight *APC* variants found in those adenomas were also considered in these analyses.

For the investigation of somatic *APC* variants, previously published results on sporadic adenomas (HGCA) were examined [20], and available in-house sequencing data generated from adenomas obtained from patients with unexplained colorectal adenomatous polyposis were re-analysed [19]. In addition, data on pathogenic germline *APC* variants were accessed from the *APC* LSDB (*www*.*lovd*.*nl/APC*).

**Fig 3:**
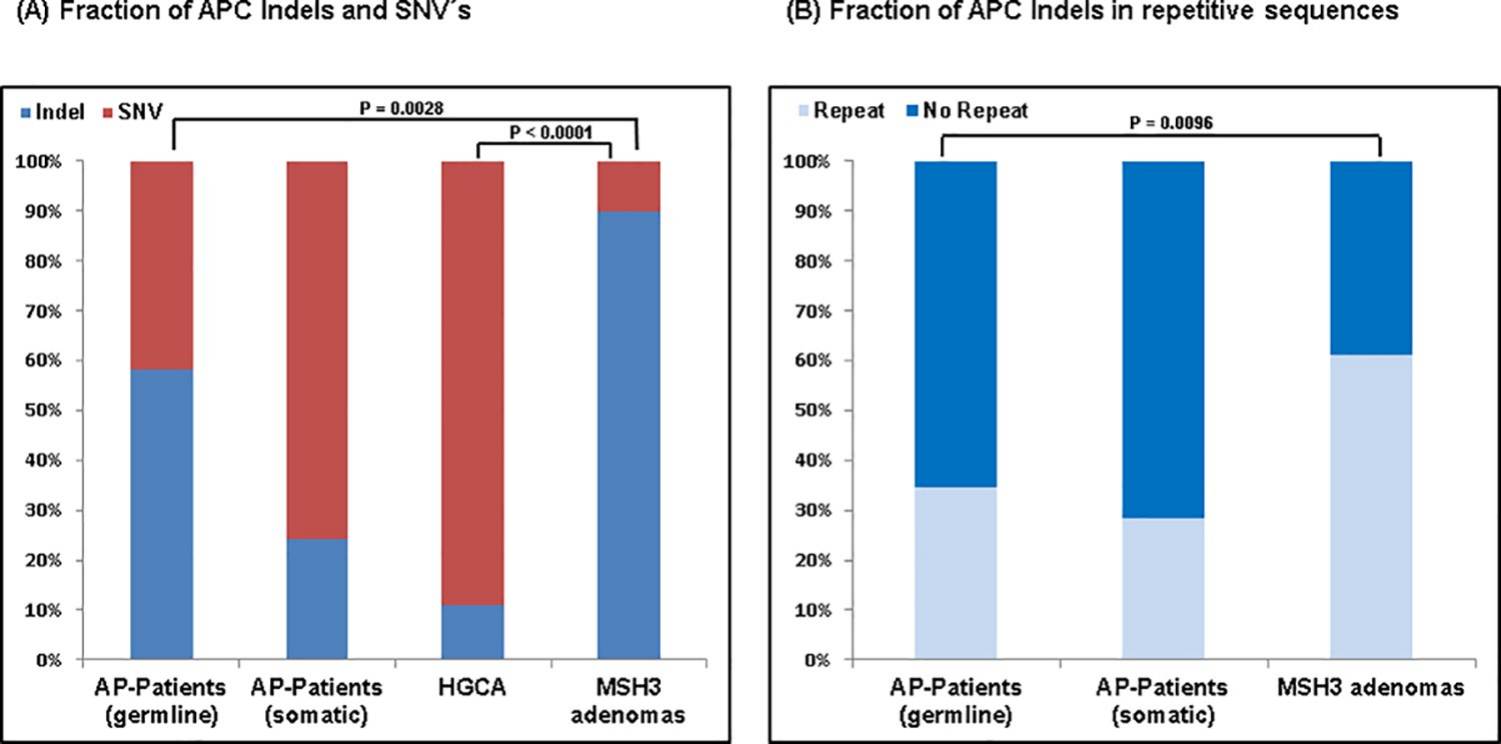
Analysis and comparison of *APC* variants in MSH3-deficient adenomas and other sources. **(A)** For *APC* variants, the proportion of indels was calculated for: patients with germline *APC* mutations (58%), termed AP patients (germline); adenomas from patients with unexplained colorectal adenomatous polyposis (24%), termed AP patients (somatic); sporadic adenomas, termed HGCA (11%); and MSH3-deficient adenomas (90%). The proportion of indels in MSH3-deficient adenomas was significantly higher than in AP patients (germline) (p = 0.0028) and in HGCA (p < 0.0001). (**B)** Of all *APC* indels, the proportion lying in a repetitive sequence context was 35% for AP patients (germline), 29% for AP patients (somatic), and 61% for MSH3-deficient adenomas. The proportion of *APC* indels in repetitive sequences was significantly higher in MSH3-deficient adenomas compared to AP patients (germline) (p = 0.0096). AP = adenomatous polyposis. HGCA = high-grade colon adenoma. Indel = insertion/deletion. SNV = Single Nucleotide Variant. Repeat = repetitive sequence.

The proportion of *APC* indels was significantly higher in the MSH3-deficient adenomas than that observed in FAP patients (p = 0.003) or in published data on sporadic adenomas (HGCA) (p < 0.0001), and was higher than in adenomas from patients with unexplained polyposis, although the latter did not reach statistical significance, probably due to low numbers of *APC* variants (Fig 3A). In addition, analysis of the repeat sequence context showed that the *APC* indels in the MSH3-deficient adenomas occurred significantly more frequently in a repetitive sequence context than *APC* indels in adenomas from patients with FAP (p < 0.01), and clearly more frequently than *APC* indels in adenomas from patients with unexplained polyposis (Fig 3B). Since the two *APC* indels in the HGCA were actually the same variant, they were not included in the repetitive sequence context analysis.

### Further (candidate) driver genes

**Fig 4:**
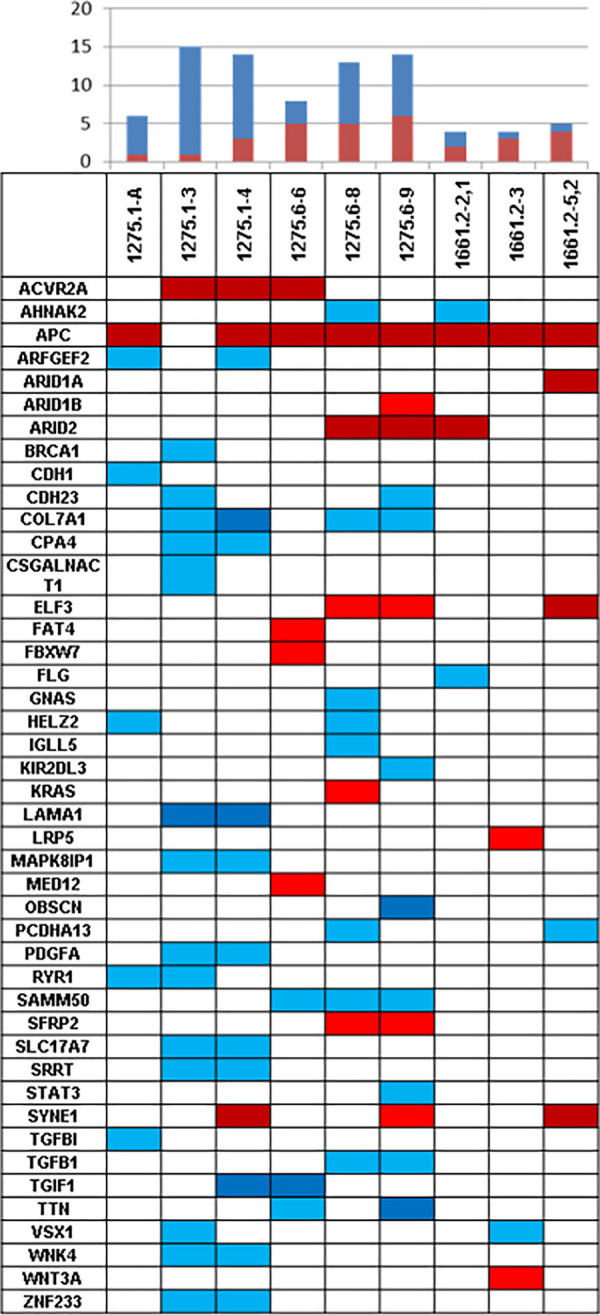
Distribution of affected (candidate) driver genes of MSH3-related tumourigenesis across all nine adenomas. The most interesting genes (Table 2) are highlighted in red, the remaining genes (see S1 Table), are highlighted in blue. The upper bar chart illustrates the number of (interesting) genes mutated per adenoma. Dark red/blue: truncating variants; light red/blue: missense variants or in-frame indels.

Filtering was performed for genes with somatic mutations in more than one adenoma, and for genes with two or more variants in one adenoma. In addition, established and published cancer driver genes mutated in at least one MSH3-deficient adenoma were selected. This approach resulted in 44 genes (Fig 4; S1 Table). On the basis of published data and biological features of relevance to tumourigenesis, 14 of these were considered interesting candidate driver genes for adenoma formation (Table 2, Fig 4, red colour). Besides *APC*, which is the main driver gene of colorectal tumourigenesis, the genes *ACVR2A* and *ARID2* were mutated in more than one polyp and are listed as Cancer Gene Census Tier 1.

Eight of the 44 genes had two or more non-silent variants in the same adenoma, suggesting that both alleles were affected (S1 Table). Five of these were considered less relevant, as based on the positions of the variants in cis or protein function and on published data. The remaining genes (*ACVR2A*, *APC*, *SYNE1*) were already considered candidate genes due to the presence of recurrent variants in more than one polyp (Table 2).

In addition, several somatic variants in established cancer driver genes were identified, including driver genes of colorectal tumourigenesis, such as *KRAS* and *FBXW7*. However, each occurred in only one adenoma (S1 Table, Fig 4).

In the three fresh-frozen adenomas, genomewide CNV analysis revealed 33 large heterozygous somatic deletions, containing 37 protein coding genes (S4 Table). However, combined with the somatic variants in all MSH3-deficient adenomas, none of the affected genes were recurrently mutated, and none were considered promising candidate genes on the basis of known function or published data.

### Pathway analysis

Pathway analysis of all genes affected by truncating variants, in-frame indels with an MAF < 0.1, and potentially pathogenic missense variants revealed an overrepresentation of genes in 25 pathways (p-value < 0.05). On the basis of the involved genes and subpathways, nine (sub)pathways were considered to be of interest (S2 Table).

## Discussion

Recently, we identified two unrelated families with attenuated colorectal adenomatous polyposis, caused by different compound-heterozygous truncating germline variants in the MMR gene *MSH3* [11]. The MMR system is a crucial pathway that corrects base-base and indel mispairs arising as a result of replication errors [29]. Since microsatellites are prone to replication errors, defects in the MMR system result in a mutator phenotype, which manifests as instability of mono-, di-, or longer nucleotide repeats in the DNA of affected cells.

During DNA repair, mispaired bases are recognised by two heterodimers of MutS homologues: MSH2 and MSH6 (MutSα); and MSH2 and MSH3 (MutSβ). MutSβ has a strong affinity for larger base-indel loops with up to ten unpaired nucleotides [30].

Previous studies have analysed the presence of MSI and the overall mutation frequency caused by MSH3 deficiency in diverse model systems. In yeast and human colon cancer cells, loss of MutSβ secondary to MSH3 inactivation results in MSI of dinucleotide repeats (usually as low MSI) and MSI of tetranucleotide repeats (EMAST) with no apparent effect on mononucleotide repeats [30–32] which is consistent with the findings in adenoma-derived DNA of the *MSH3*-related polyposis patients [11].

### Spectrum of somatic variants in MSH3-deficient adenomas

The mean TMB (2.3 variants/Mb) in the present study was similar to that observed in previous WES analyses of sporadic (0.6–4.6 variants/Mb) and FAP-related (0.2–1.8 variants/Mb) MSS colon adenomas, although the published TMB showed wide variability, probably due to cross-study differences in adenoma size, methodology, and filter criteria [20, 33–38]. This is in the same order of magnitude as the average TMB in non-hypermutated CRC (2.8–4.3 variants/Mb) found in other studies and in data from the Cancer Genome Atlas [34, 37]. The results are also in line with mouse models [39–41].

There are some possible reasons for this observation. Given the functional mechanism of MSH3, MSH3 deficient tumours are expected to show neither an increased, hypermutated rate of SBS, nor any specific type of SBS. Especially in coding regions there are only few typical target sequences (EMAST sequences) that might be subject of MSH3-related repair as e.g. only seven tetranucleotide repeats in predicted gene-coding regions were retrieved in a genome-wide database search [42]. In addition, adenomas in general do not show a high number of somatic variants and a striking overall lead of SBS so that even a reduced DNA repair of indels might not result in recognisable differences at this stage of tumourigenesis.

In contrast, the TMB of MSI CRCs (35–51 variants/Mb, TCGA data >12 variants/Mb) or Lynch syndrome associated adenomas, respectively, is an order of magnitude higher [34, 37, 43, 44]. Comparison of these results with data from CMMRD-associated adenomas would be interesting, but few such data are available [45].

The predominant variant type in the present MSH3-deficient adenomas (C:G>T:A transitions) is consistent with that reported in WES studies of sporadic and FAP-related colon adenomas [33, 35, 37]. Nonspecific SBS signature 1, which is characterised by C>T transitions at NpCpG trinucleotides, is the most frequent signature across all cancer classes and is mainly caused by an age-related increase in the spontaneous deamination of 5-methyl-cytosine (COSMIC). Signature 1 contributes similar numbers of variants to most cancer types, and may represent a cell division / mitotic clock. In contrast, SBS 6 is associated with defective MMR and found in microsatellite unstable tumours (COSMIC). The lack of further more specific mutational SBS signatures is consistent with impaired MSH3 function, which affects indels rather than SBS. However, due to the relatively small number of somatic SBS in the MSH3-deficient adenomas, the possibility of additional signatures in *MSH3*-related CRC cannot be excluded.

In line with the expected mutation type, we identified a significant increase of indels in MSH3-deficient adenomas compared to HGCA, including a substantial number of 4 bp deletions. The majority of indels were located within a repeat context, which is in accordance with the observation that mismatches are more likely to occur within repeat sequences. Interestingly, the fraction of 1 bp deletions in MSH3-deficient adenomas was considerably lower than that reported in HGCA, suggesting the involvement of differing mutational mechanisms.

Published indel frequencies on MMR-deficient adenomas are scarce. Interestingly, the fraction of indels in MSH3-deficient adenomas is similar to the one extracted from the study of Kim et al. [43], which analysed WES data from four advanced sporadic MSI-H adenomas. However, the data by Kim et al. show a striking variability across adenomas and between adenoma and CRC tissue and the results are difficult to compare due to different methods and study designs and the small number of tumours included.

Tetranucleotide indels were not the predominant mutation type in coding regions of the present MSH3-deficient adenomas and the majority of the tetranucleotide indels were not located within obvious repeat sequences, although the adenomas showed high instability at di- and tretranucleotide repeats in noncoding regions, as previously demonstrated [11]. This might be attributable to the low number of somatic variants—including indels—in adenomas in general compared to advanced cancers, and in particular the relatively low number of potential tetranucleotide repeat targets throughout the genome and in protein coding genes [42].

Interestingly, small ID signatures ID2 and ID4 were found in all nine polyps. ID2 is found in most types of cancer, and a substantial number of variants with this signature has been reported in MMR-deficient / hypermutated malignancies [24]. In addition, this signature has been observed in non-hypermutated cancers associated with SBS1 (COSMIC). The aetiology of ID4, which represented > 60% of the ID signatures in each of the nine polyps, remains unknown. ID4 shows large numbers of mutations in a subset of samples, which do not obviously have the mutational features of defective DNA MMR and thus, might be a signature not specific for MMR deficiency (COSMIC). Notably, ID4 has not been found in CRC samples and seems to be rare in tumours in general [24].

Based on these observations, the effect of checkpoint blockade in MSH3-deficient tumours is difficult to predict. Since frameshift peptides are a driving force of efficient checkpoint immune therapy, the expected higher number of indels in MSH3-related CRC might result in a benefit, but the effect would probably be lower than in classic MSI tumours. However, the preliminary data obtained in adenomas do not allow such conclusions.

### Somatic *APC* variants

A key finding of the present study was that almost all (8/9) of the MSH3-deficient adenomas harboured truncating somatic *APC* variants as the most relevant driver mutation that is supposed to initiate colorectal tumour formation. In five of the nine adenomas, two *APC* variants were found. This indicates a biallelic inactivation of the gene, which is in line with its known function as a TSG. In addition, the number of variants in the *APC* MCR was similar to that observed in sporadic adenomas which points to the oncogenic relevance of the MCR for tumourigenesis irrespective of the underlying mutation type. These findings indicate that *APC* variants are the initial driving force of adenoma formation in *MSH3*-patients and thus, *MSH3*-related tumours mainly follow the classical Wnt signaling pathway of colorectal tumourigenesis. This observation is consistent with findings in tumours from other inherited adenomatous polyposis syndromes arising secondary to pathogenic germline variants in DNA repair genes, such as MAP, PPAP, and NAP. An interesting approach would be to compare these data with the impact and spectrum of somatic *APC* variants in adenomas derived from patients with CMMRD. To our knowledge, however, no such data are yet available.

Interestingly, the pattern of somatic *APC* variants reflects impaired MSH3 repair function, since all but one of the truncating APC variants consisted of 2-, 4-, or 5 bp deletions. In addition, the fraction of indels among all somatic APC variants (90%) and the fraction of *APC* indels in repetitive sequences was significantly higher in the MSH3-deficient adenomas. This mutational pattern was not found in any of the other candidate driver genes, and might be attributable to the specific sequence composition of *APC*, which qualifies the gene as a favoured tumour driver target of deficient MSH3 repair. In a previous study of FAP-related colorectal adenomas, only 3/13 (23%) truncating somatic *APC* variants were indels [37]. These findings indicate that the majority of somatic *APC* variants in MSH3-deficient adenomas are present due to impaired MSH3 function.

These findings are in line with mice studies: Compared to *Apc*^1638N^ mice deficient for MSH6, which predominantly showed somatic base-pair substitutions of the wild type *APC* allele (just 5 of 76 variants were indels), most of the *APC* variants in MSH3-deficient *Apc*^1638N^ mice (5/7) were frameshift variants, and of these, three were dinucleotide insertion/deletions that were not found in other MMR-deficient Apc^1638N^ tumours, and two were large (≥8 bp) deletions [46].

### Candidate driver genes

While variants in established cancer driver genes were identified in several of the present MSH3-deficient adenomas, each of the known more specific drivers of colorectal tumourigenesis (*KRAS*, *FBWX7*) was affected in only a single adenoma with the exception of *APC*. No somatic variants were found in *TP53*, *PIK3CA*, or *SMAD4*, which are usually mutated in advanced stages of tumourigenesis (CRC).

Thirtheen genes (*ACVR2A*, *ARID1A*, *ARID1B*, *ARID2*, *ELF3*, *FAT4*, *FBXW7*, *KRAS*, *LRP5*, *MED12*, *SFRP2*, *SYNE1*, and *WNT3A*) were considered promising potential candidate driver genes of early *MSH3*-related tumourigenesis on the basis of the presence of recurrent variants and/or their biological function and involvement in molecular processes of relevance to tumourigenesis. None of these genes showed out-of-frame polynucleotide indels. Thus rather than being a direct target of impaired MSH3 function, they may be subject to other mutational mechanisms that develop during tumourigenesis.

Among the most interesting driver genes identified in the present MSH3-deficient adenomas are *ACVR2A* and *ARID2*. *ACVR2A* is a known TSG in CRC, which mediates the functions of members of the TGFß superfamily [47, 48]. Since it harbours coding microsatellites similar to *TGFBR2*, *ACVR2A* is one of the most frequently mutated genes in CRC in patients with Lynch syndrome [49, 50] and perturbation of TGFß signalling through truncating variants in *ACVR2A* is suggested to be an early event in CRC carcinogenesis [43]. Consistent with this, *ACVR2A* is regarded as a driver gene in MSI colorectal adenomas and is—after *TGFBR2*—the gene with the second highest rate of frameshift variants in MSI adenomas [43, 51]. In line with this, we identified the same 1 bp deletion in the 8-bp polyadenine [(A)8] tract in exon 10 of the *ACVR2A* gene in three adenomas (Table 2), which is a frequent microsatellite indel driver hotspot in colorectal MSI neoplasms [52–54].

Further promising driver genes affected in four different MSH3-deficient adenomas, are the *ARID* genes (Table 2, S1 Table). It was shown, that 13% of MSI CRC carried *ARID2* variants [55] and studies of intestinal tumour organoids and sporadic colorectal adenomas indicate that *ARID2* may function as a TSG [20, 56]. As with *ARID2*, the TSG and candidate drivers of colorectal adenomas *ARID1A*, and *ARID1B* are frequently mutated in MSI tumours including adenomas [37, 43, 55, 57, 58]. *ARID* genes also seem to be connected to *RUNX1 (S2 Table)*.

The present analyses detected further recurrently mutated genes, which are proposed candidate drivers of colorectal adenomas (S1 Table) such as *OBSCN* [34] or the TSG *SYNE1* whose promotor was methylated in all, and mutated in several CRC cases [36, 59, 60]. In addition, we found variants in genes implicated in Wnt signalling, such as *LRP5*, *WNT3A*, and *SFRP2* (Table 2), which shows a significantly increased level of methylation in adenomas [61, 62]. For other genes, discussed as potential early drivers of colorectal tumourigenesis (S1 Table) published data are conflicting, limited, or weak, and thus, further evidence is required before their role as relevant drivers can be evaluated.

Taken together, besides *APC*, the nine adenomas harboured between one to five further variants in established or likely driver genes (Fig 4, red colour). Interestingly, variants in *ACVR2A* and in the *ARID* genes occurred in 7/9 adenomas. These data suggest that with the exception of *ARID1B*, mutations in *ACVR2A* or an *ARID* gene were mutually exclusive. However, *ARID1B* was affected by an inframe deletion that might not be deleterious.

Although *APC* variants are the predominant drivers in hereditary adenomatous polyposis syndromes, the somatic variant type and spectrum reflect the aetiology of the mutational process, i.e. the underlying gene that is impaired by a germline pathogenic variant. In contrast to the enrichment of G:C>T:A transversions in MAP, C:G>T:A transitions in NAP, or SBS in PPAP [5, 6, 63, 64], *MSH3*-related colorectal tumours are characterised by a predominance of small, out-of-frame deletions across the *APC* gene, further indicating that the mutation profile can provide a hint as to the underlying germline defect. Hence, mutational profiles generated by routine tumour sequencing might be particularly helpful in terms of identifying very rare hereditary tumour syndromes, such as *MSH3*-related adenomatous polyposis. In addition, specific profiles can support the assignment of rare extraintestinal lesions to the tumour spectrum of a novel—and as yet insufficiently described—tumour syndrome, as demonstrated recently for NAP [9].

The present study has some limitations. It would have been very interesting to include carcinoma tissue in the study to compare the variant burden and spectrum between early and advanced MSH3-deficient tumours and other MSI- and MSS CRCs, and to identify potential driver genes, relevant for advanced steps of tumourigenesis. Unfortunately, no malignant tissue was available from the few MSH3-related polyposis patients.

Since the majority of polyps was archived material, FFPE-induced sequencing artifacts cannot be ruled out completely. However, we used methods to correct for those artifacts and never experienced any major problem from FFPE samples in the past [16, 65]. In addition, we compared the results of FFPE polyps (1275.1, 1275.6) with fresh frozen polyps (1661.2 and Lee et al. 2017) and found a similar amount of C>T substitutions (S2 Fig). Therefore, we estimate an FFPE-induced bias as being small, although we cannot exclude a slight increase of false positive findings.

It is well known that the spectrum and prevalence of specific driver mutations and (consensus) molecular subtypes of CRC differ by site (right/left) and location [66]. However, according to our knowledge, no such data on adenomas are available. In the present study, the numbers of adenomas with known exact location per patient was too small to investigate further interesting genetic aspects of colorectal tumours such as varying variant pattern, variant numbers, or EMAST across anatomical regions.

In summary, the present data suggest that compared to sporadic adenomas (HGCA), the somatic mutation spectrum of MSH3-deficient adenomas is characterised by a general increase in the number of indels and a more specific pattern of somatic variants. The latter include out-of-frame polynucleotide deletions in the *APC* gene as initial driver of adenoma formation. Given its relevance as gatekeeper of colorectal tumourigenesis, APC is likely to be the main tumourigenesis-relevant target of MSH3 deficiency. Besides established driver genes of colorectal tumourigenesis, our data suggest that *ACVR2A* and the *ARID* genes in particular are important targets in terms of adenoma formation. The findings of this study need to be validated in larger series including malignant tissue and polyps with different grades of dysplasia.

## Supporting information

S1 Fig Pedigrees of the two independent families with Bilallelic Germline *MSH3*-Mutations. Arrows indicate patients included in the present investigation. Identifiers for affected individuals are shown above the respective symbols. The number on the upper right side of a symbol indicates age at death, or in living persons, the age at last contact. Phenotype information is displayed on the bottom left. The numbers following a disease represent the age at first diagnosis. ad. = adenomas. CRC = colorectal carcinoma. duod. = duodenal GC = gastric cancer. Polyposis = multiple colorectal adenomatous polyps. yrs = years.
(TIF)

S2 Fig Single nucleotide variant profile of MSH3-deficient adenomas and sporadic adenomas (HGCA). SNVs are classified according to sequence changes. Relative proportions of sequence based variant categories (y-axis) are shown for each polyp. (**A)** MSH3-deficient adenomas including silent variants. (**B)** For comparison, HGCAs are shown, silent variants were excluded (from Lee et al., 2017, copied with permission).
(TIF)

S3 Fig 1–9 single base substitution mutational signatures of all nine polyps using deconstructSigs. The top panel represents the mutational profile of the polyp and displays the proportion of mutations found in each trinucleotide context. The middle panel shows the reconstructed mutational profile created using deconstructSigs and by multiplying the calculated weights by the signatures. The bottom panel shows the error (SSE = sum-squared error) between the tumour mutational profile and the reconstructed mutational profile.
(ZIP)

S4 Fig Variant features in MSH3-deficient adenomas and sporadic adenomas (HGCA). **A)** The number of non-silent somatic variants in the nine MSH3-deficient adenomas are shown according to five functional categories, as indicated in the insets. **B)** For comparison, the mutational features of eleven HGCA are displayed (from Lee et al., 2017, copied with permission). FS = frameshift. IF = inframe. Indel = insertion/deletion.
(TIF)

S5 Fig Indel pattern in MSH3-deficient adenomas and sporadic adenomas (HGCA). The number of inserted or deleted nucleotides is shown on the x-axis (e.g. Indel-1 = mononucleotides, Indel-2 = dinucleotides); the absolute numbers of corresponding indel variants in all MSH3-deficient adenomas and HGCA (data based on Lee et al., 2017) are shown on the y-axis. The proportion of indels lying in repetitive sequences is displayed in light blue and light red (Repeat).
(TIF)

S6 Fig Variant features of MSH3-deficient adenomas including silent variants. For the nine MSH3-deficient adenomas, the proportion of all somatic variants, including silent variants, is shown in accordance with six functional categories. FS = frameshift. IF = inframe. Indel = insertion/deletion.
(TIF)

S7 Fig Variant c.1303delA in the gene *ACVR2A*. As an example, the 1 bp deletion c.1303delA in *ACVR2A* within a mononucleotide repeat in polyp 1275.1–3 is depicted (Screenshot of the Integrative Genomics Viewer, not all reads are shown).
(TIF)

S1 Table Somatic variants of (candidate) driver genes in MSH3-deficient adenomas. The variants in the MSH3-deficient adenomas were filtered for: established driver genes in colorectal cancer and adenomas, genes with recurrent variants in ≥ one adenoma, genes with ≥ one variant in one adenoma, and genes involved in Wnt-signaling or identified through pathway analysis.
(XLSX)

S2 Table Result of pathway analysis. The most interesting pathways extracted via Reactome are shown. Ratio refers to the proportion of Reactome pathway molecules represented by this pathway. The p-value is the result of the statistical test for over-representation, and the False Discovery rate (FDR) is the corrected probability of over-representation.
(XLSX)

S3 Table Somatic variants identified in MSH3-deficient adenomas. This list contains all somatic variants identified in MSH3-deficient adenomas after the filtering process and before read check with Integrative Genome Viewer.
(XLSX)

S4 Table Somatic CNVs detected in polyps from patient 1661.2. This list contains the 33 CNVs identified in polyps 1661.2–2.1, 1661.2–3 and 1661.2–5.2 after the filtering process.
(XLSX)
