# Supplementary figures and images for: Variant profiling of colorectal adenomas from three patients of two families with MSH3-related adenomatous polyposis
Source: PLoS One. 2021 Nov 29;16(11):e0259185. doi: 10.1371/journal.pone.0259185 (PMC8629245; doi:10.1371/journal.pone.0259185)

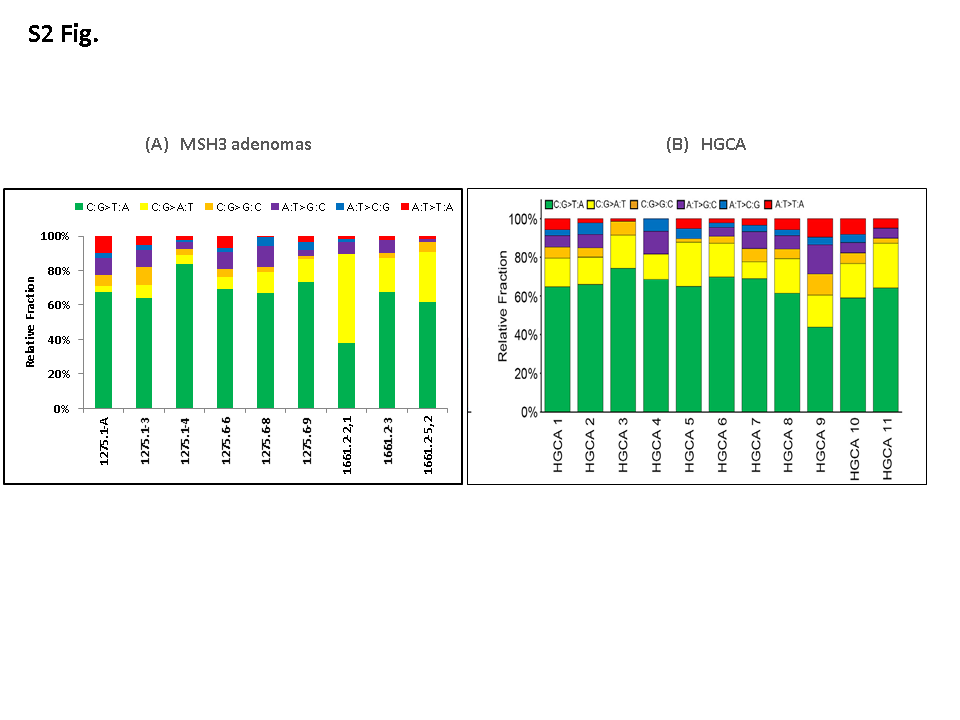

Supplement: S2 Fig — SNVs are classified according to sequence changes. Relative proportions of sequence based variant categories (y-axis) are shown for each polyp. (A) MSH3-deficient adenomas including silent variants. (B) For comparison, HGCAs are shown, silent variants were excluded (from Lee et al., 2017, copied with permission). (TIF) [file pone.0259185.s002.tif]

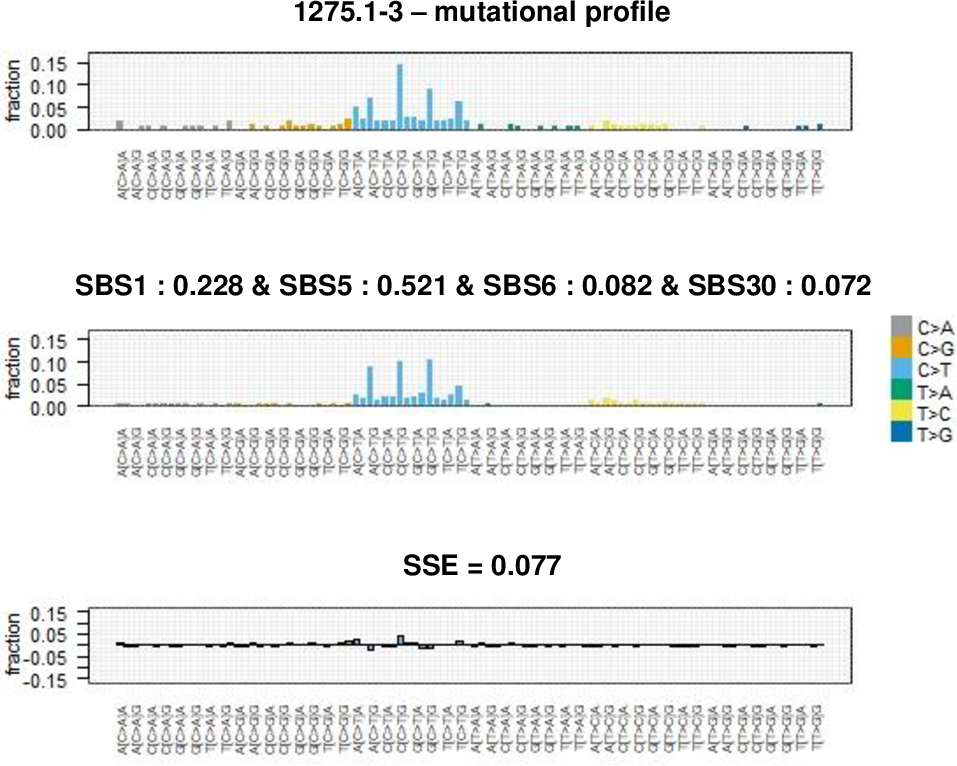

Supplement: S3 Fig — The top panel represents the mutational profile of the polyp and displays the proportion of mutations found in each trinucleotide context. The middle panel shows the reconstructed mutational profile created using deconstructSigs and by multiplying the calculated weights by the signatures. The bottom panel shows the error (SSE = sum-squared error) between the tumour mutational profile and the reconstructed mutational profile. (ZIP) [file pone.0259185.s003.zip › S3_Fig_1.tif]

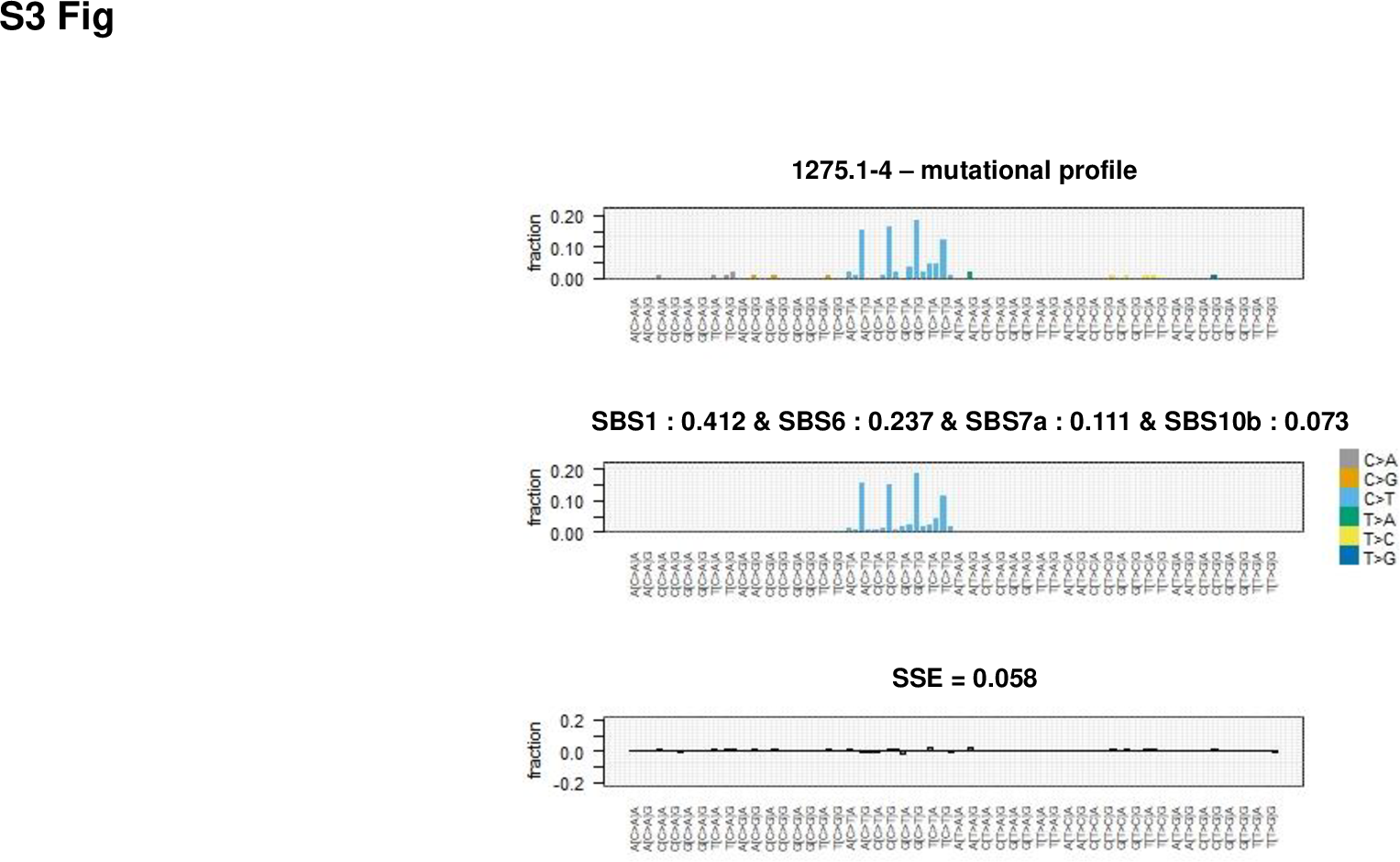

Supplement: S3 Fig — The top panel represents the mutational profile of the polyp and displays the proportion of mutations found in each trinucleotide context. The middle panel shows the reconstructed mutational profile created using deconstructSigs and by multiplying the calculated weights by the signatures. The bottom panel shows the error (SSE = sum-squared error) between the tumour mutational profile and the reconstructed mutational profile. (ZIP) [file pone.0259185.s003.zip › S3_Fig_2.tif]

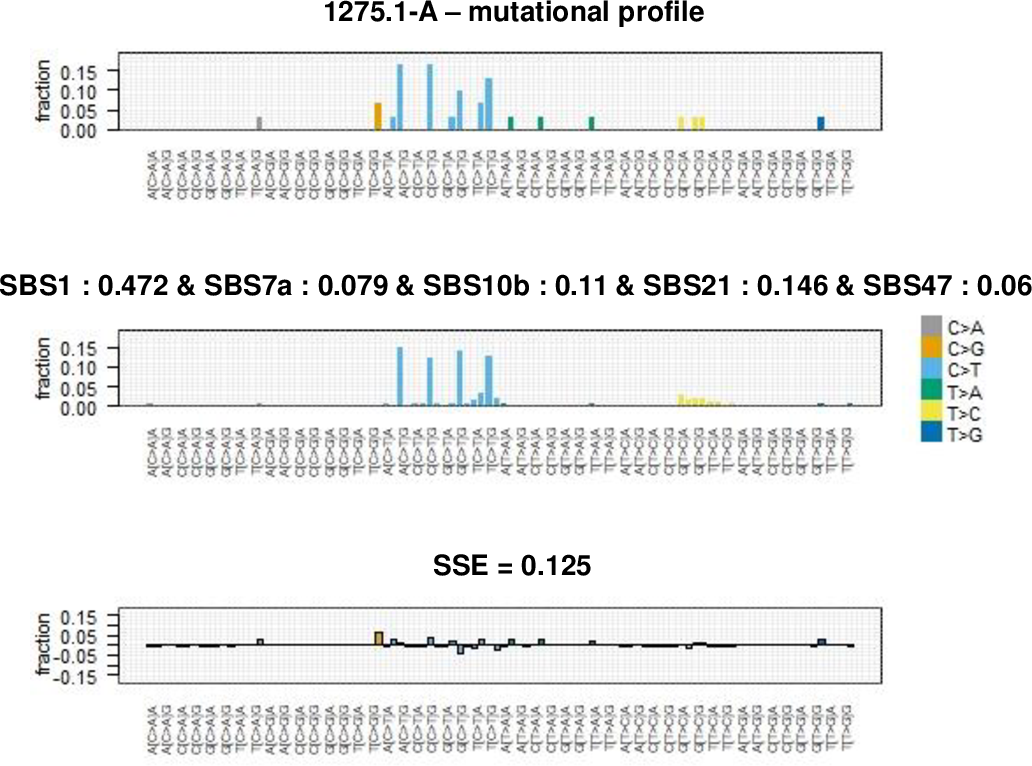

Supplement: S3 Fig — The top panel represents the mutational profile of the polyp and displays the proportion of mutations found in each trinucleotide context. The middle panel shows the reconstructed mutational profile created using deconstructSigs and by multiplying the calculated weights by the signatures. The bottom panel shows the error (SSE = sum-squared error) between the tumour mutational profile and the reconstructed mutational profile. (ZIP) [file pone.0259185.s003.zip › S3_Fig_3.tif]

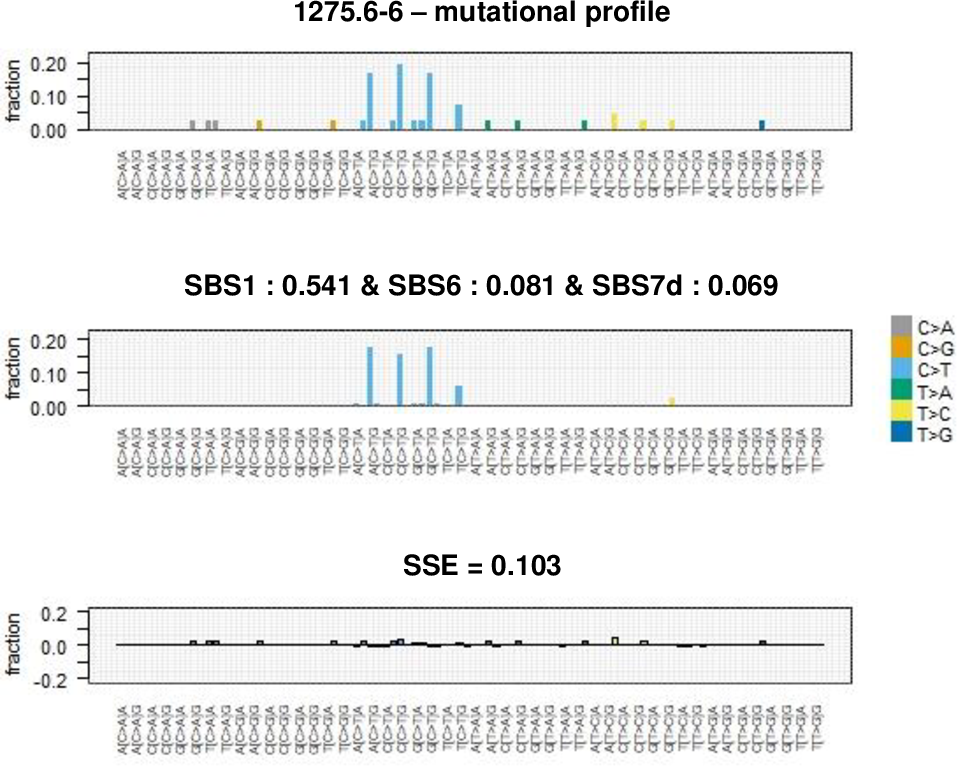

Supplement: S3 Fig — The top panel represents the mutational profile of the polyp and displays the proportion of mutations found in each trinucleotide context. The middle panel shows the reconstructed mutational profile created using deconstructSigs and by multiplying the calculated weights by the signatures. The bottom panel shows the error (SSE = sum-squared error) between the tumour mutational profile and the reconstructed mutational profile. (ZIP) [file pone.0259185.s003.zip › S3_Fig_4.tif]

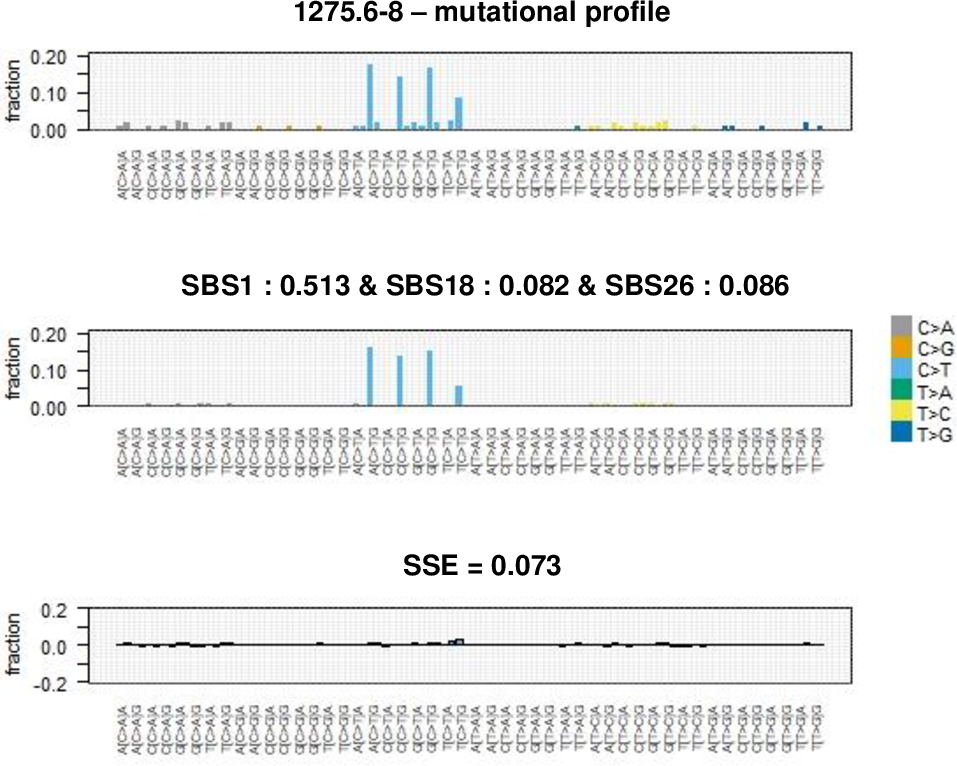

Supplement: S3 Fig — The top panel represents the mutational profile of the polyp and displays the proportion of mutations found in each trinucleotide context. The middle panel shows the reconstructed mutational profile created using deconstructSigs and by multiplying the calculated weights by the signatures. The bottom panel shows the error (SSE = sum-squared error) between the tumour mutational profile and the reconstructed mutational profile. (ZIP) [file pone.0259185.s003.zip › S3_Fig_5.tif]

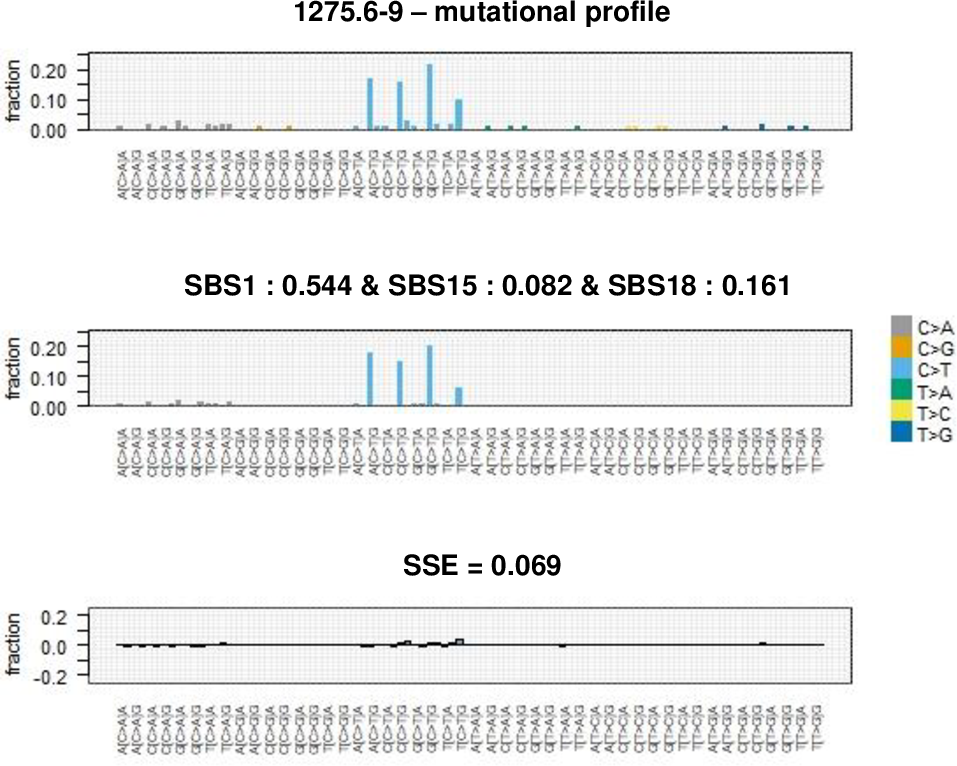

Supplement: S3 Fig — The top panel represents the mutational profile of the polyp and displays the proportion of mutations found in each trinucleotide context. The middle panel shows the reconstructed mutational profile created using deconstructSigs and by multiplying the calculated weights by the signatures. The bottom panel shows the error (SSE = sum-squared error) between the tumour mutational profile and the reconstructed mutational profile. (ZIP) [file pone.0259185.s003.zip › S3_Fig_6.tif]

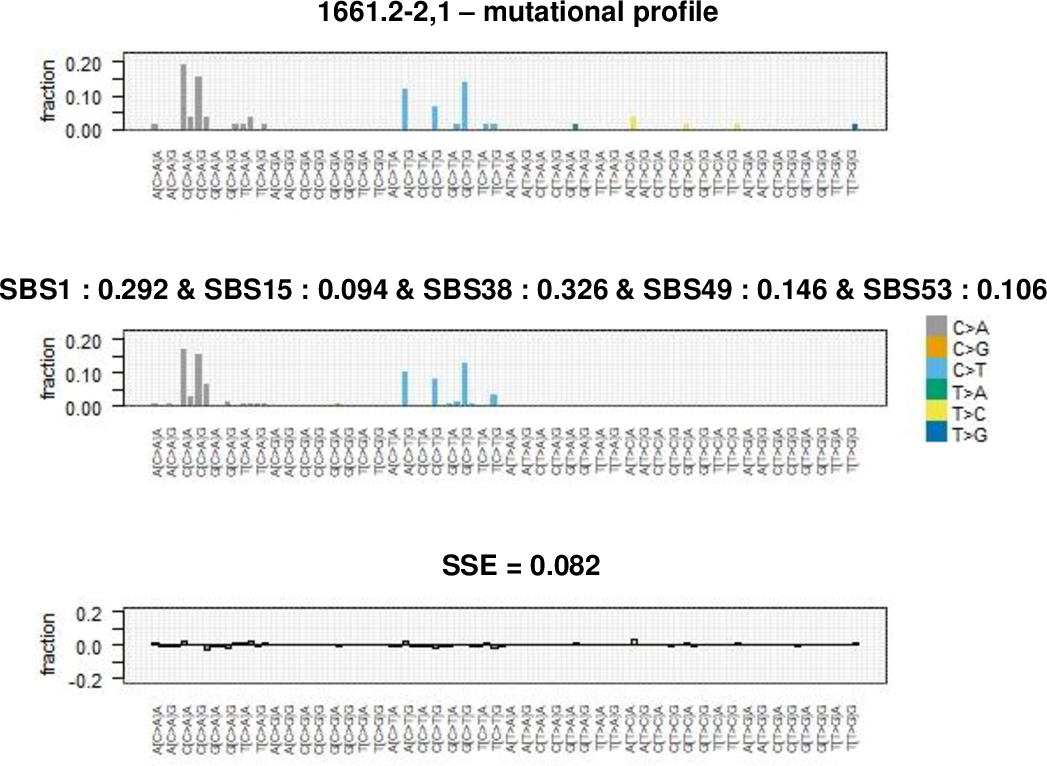

Supplement: S3 Fig — The top panel represents the mutational profile of the polyp and displays the proportion of mutations found in each trinucleotide context. The middle panel shows the reconstructed mutational profile created using deconstructSigs and by multiplying the calculated weights by the signatures. The bottom panel shows the error (SSE = sum-squared error) between the tumour mutational profile and the reconstructed mutational profile. (ZIP) [file pone.0259185.s003.zip › S3_Fig_7.tif]

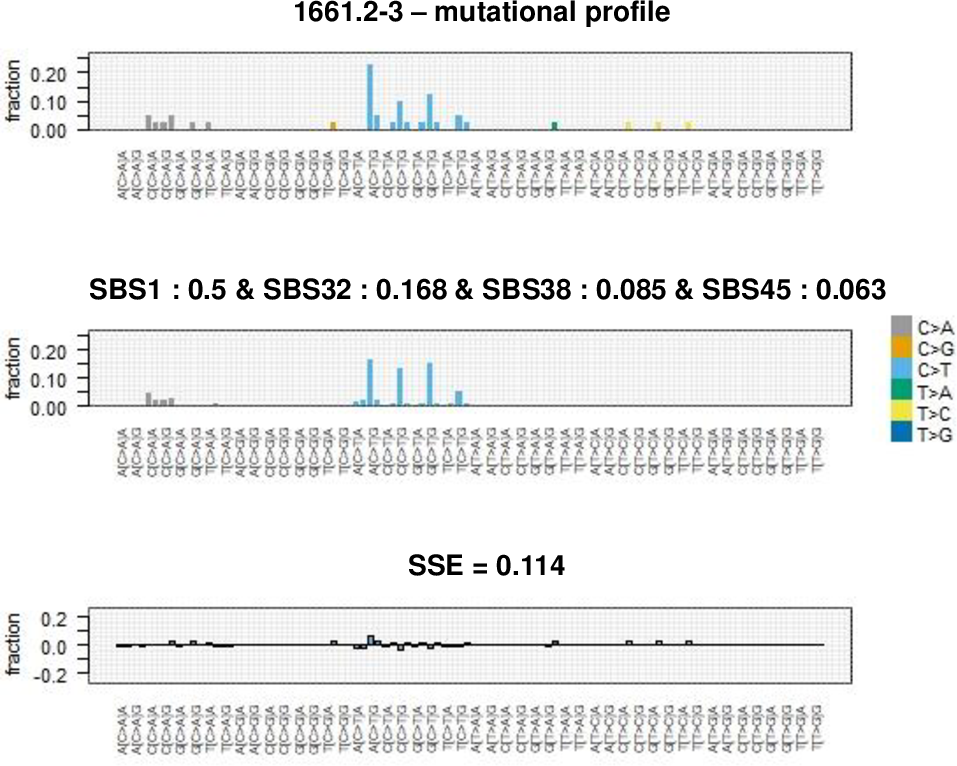

Supplement: S3 Fig — The top panel represents the mutational profile of the polyp and displays the proportion of mutations found in each trinucleotide context. The middle panel shows the reconstructed mutational profile created using deconstructSigs and by multiplying the calculated weights by the signatures. The bottom panel shows the error (SSE = sum-squared error) between the tumour mutational profile and the reconstructed mutational profile. (ZIP) [file pone.0259185.s003.zip › S3_Fig_8.tif]

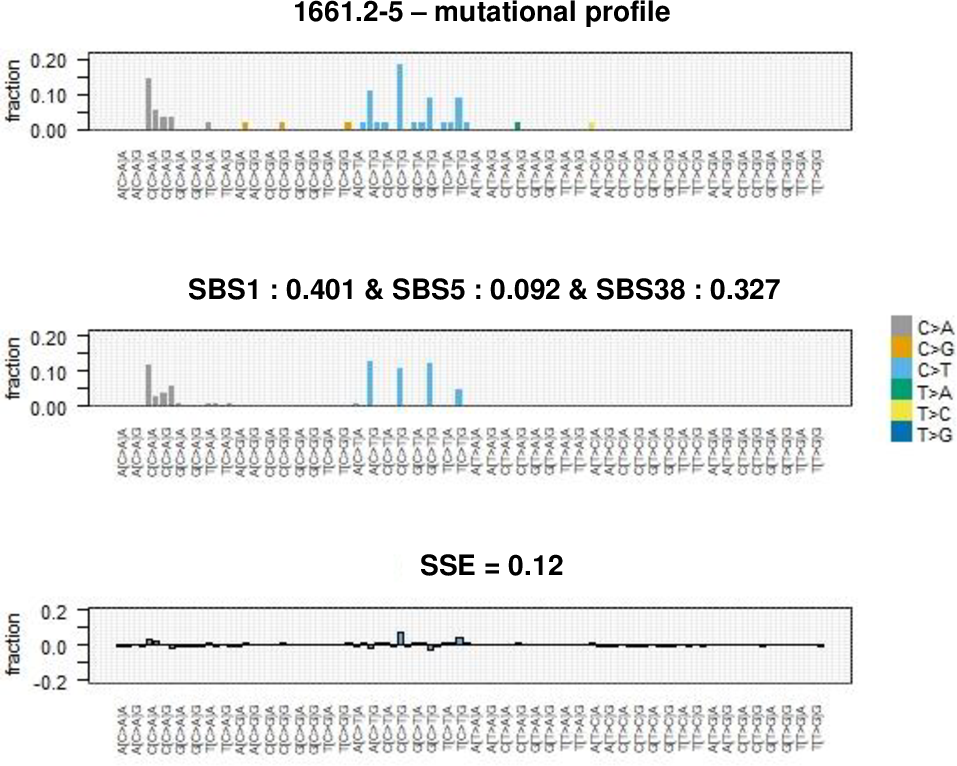

Supplement: S3 Fig — The top panel represents the mutational profile of the polyp and displays the proportion of mutations found in each trinucleotide context. The middle panel shows the reconstructed mutational profile created using deconstructSigs and by multiplying the calculated weights by the signatures. The bottom panel shows the error (SSE = sum-squared error) between the tumour mutational profile and the reconstructed mutational profile. (ZIP) [file pone.0259185.s003.zip › S3_Fig_9.tif]

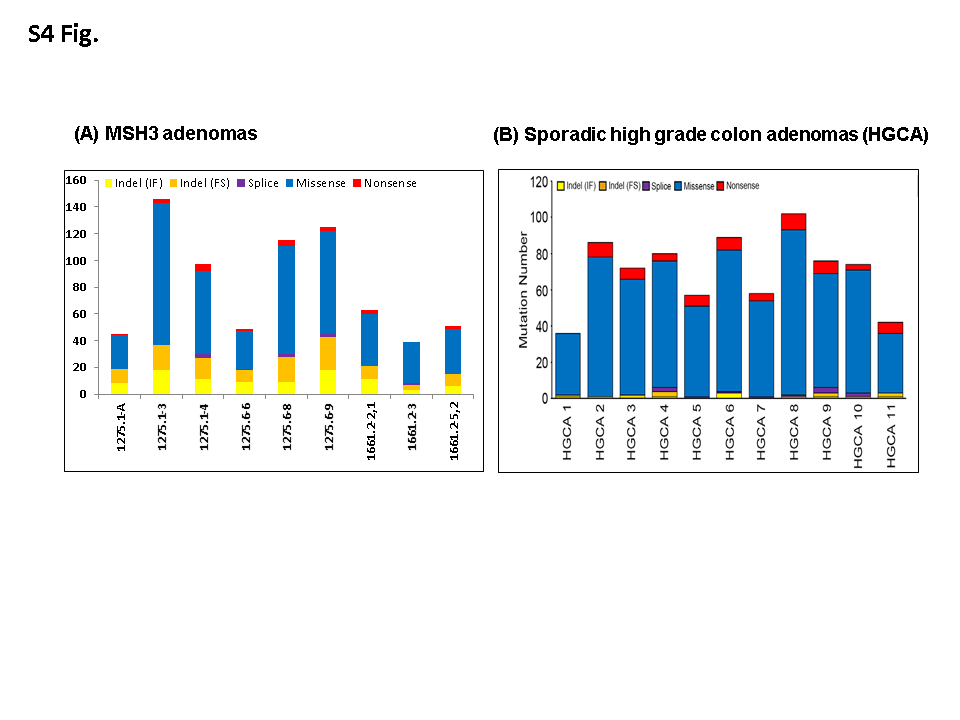

Supplement: S4 Fig — A) The number of non-silent somatic variants in the nine MSH3-deficient adenomas are shown according to five functional categories, as indicated in the insets. B) For comparison, the mutational features of eleven HGCA are displayed (from Lee et al., 2017, copied with permission). FS = frameshift. IF = inframe. Indel = insertion/deletion. (TIF) [file pone.0259185.s004.tif]

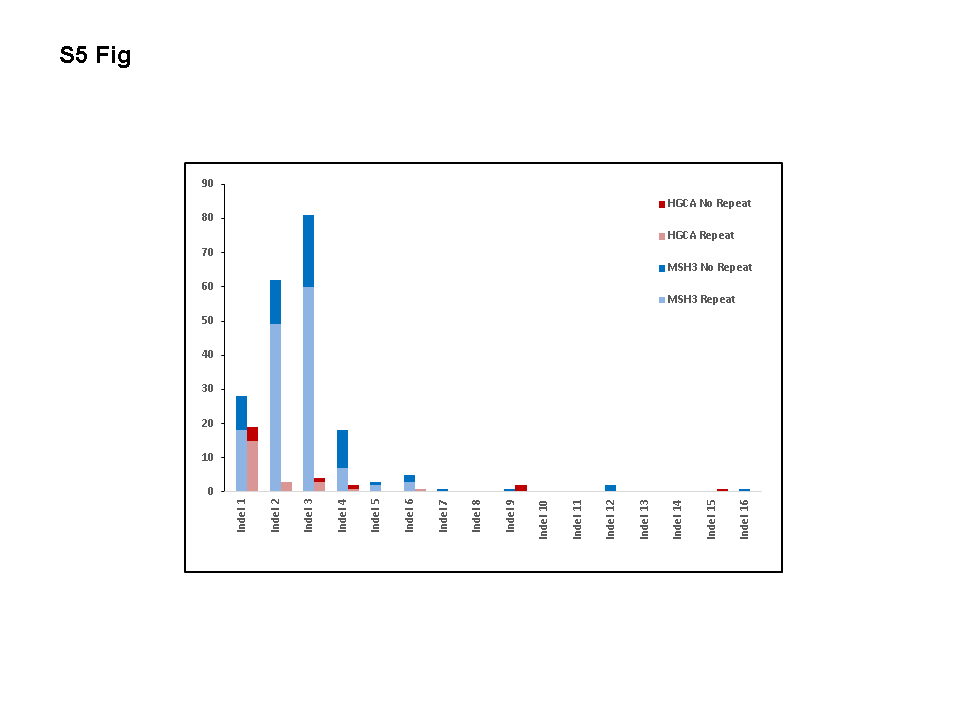

Supplement: S5 Fig — The number of inserted or deleted nucleotides is shown on the x-axis (e.g. Indel-1 = mononucleotides, Indel-2 = dinucleotides); the absolute numbers of corresponding indel variants in all MSH3-deficient adenomas and HGCA (data based on Lee et al., 2017) are shown on the y-axis. The proportion of indels lying in repetitive sequences is displayed in light blue and light red (Repeat). (TIF) [file pone.0259185.s005.tif]

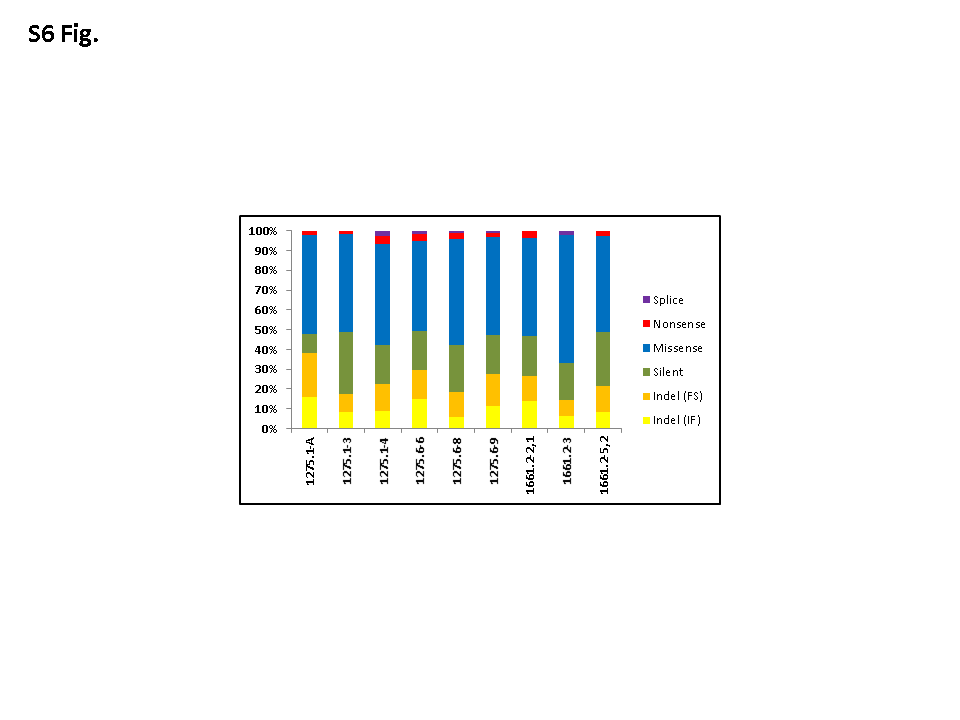

Supplement: S6 Fig — For the nine MSH3-deficient adenomas, the proportion of all somatic variants, including silent variants, is shown in accordance with six functional categories. FS = frameshift. IF = inframe. Indel = insertion/deletion. (TIF) [file pone.0259185.s006.tif]

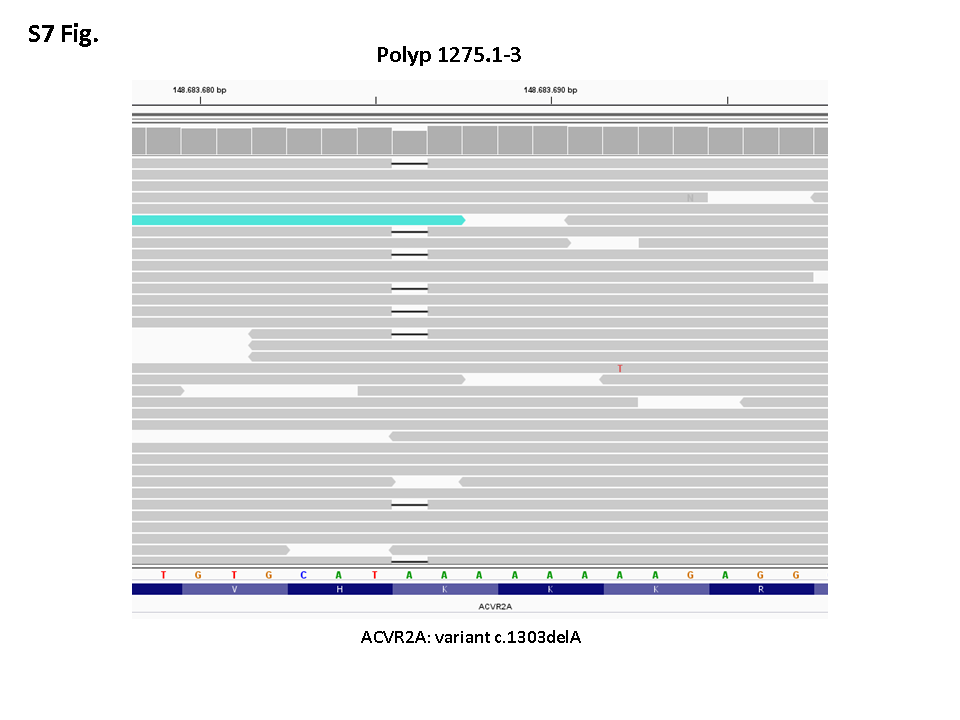

Supplement: S7 Fig — As an example, the 1 bp deletion c.1303delA in ACVR2A within a mononucleotide repeat in polyp 1275.1–3 is depicted (Screenshot of the Integrative Genomics Viewer, not all reads are shown). (TIF) [file pone.0259185.s007.tif]
